# Optimisation of region-specific reference gene selection and relative gene expression analysis methods for pre-clinical trials of Huntington's disease

**DOI:** 10.1186/1750-1326-3-17

**Published:** 2008-10-27

**Authors:** Caroline L Benn, Helen Fox, Gillian P Bates

**Affiliations:** 1 Medical and Molecular Genetics, King's College London School of Medicine, 8th Floor Tower Wing, Guy's Hospital, Great Maze Pond, London, SE1 9RT, UK

## Abstract

**Background:**

Transcriptional dysregulation is an early, key pathogenic mechanism in Huntington's disease (HD). Therefore, gene expression analyses have biomarker potential for measuring therapeutic efficacy in pre-clinical trials, particularly those aimed at correcting gene expression abnormalities. Housekeeping genes are commonly used as endogenous references in gene expression studies. However, a systematic study comparing the suitability of candidate reference genes for use in HD mouse models has not been performed. To remedy this situation, 12 housekeeping genes were examined to identify suitable reference genes for use in expression assays.

**Results:**

We found that commonly used reference genes are dysregulated at later time points in the R6/2 mouse model of HD. Therefore, in order to reliably measure gene expression changes for use as pre-clinical trial biomarkers, we set out to identify suitable reference genes for use in R6/2 mice. The expression of potential reference genes was examined in striatum, cortex and cerebellum from 15 week old R6/2 and matched wild-type littermates. Expression levels of candidate reference genes varied according to genotype and brain region. GeNorm software was used to identify the three most stably expressed genes for each brain region. Relative quantification methods using the geometric mean of three reference genes for normalisation enables accurate determination of gene expression levels in wild-type and R6/2 mouse brain regions.

**Conclusion:**

Our study has identified a reproducible, reliable method by which we able to accurately determine the relative expression level of target genes in specific brain regions, thus increasing the potential of gene expression analysis as a biomarker in HD pre-clinical trials.

## Background

Huntington's disease (HD) is an autosomal dominant, late-onset neurodegenerative disorder caused by a CAG repeat expansion in exon 1 of the *HD *gene which translates into a polyglutamine (polyQ) tract in the huntingtin (Htt) protein [1]. HD thus belongs to a group of neurodegenerative disorders caused by polyQ expansion, which include spinal and bulbar muscular atrophy (SBMA), dentatorubral pallidoluysian atrophy (DRPLA) and the spinocerebellar ataxias (SCA) types 1, 2, 3, 6, 7, and 17 [2]. The polyQ motif occurs in many transcription factors and can function as a transcriptional activation domain. Interestingly, polyQ repeat expansions in TATA binding protein (TBP) and the androgen receptor (AR) cause the disorders SCA17 and SBMA, respectively. Indeed, polyQ repeat containing disease proteins are increasingly implicated as mediators of gene expression, with deranged transcription profiles observed as a result of polyQ repeat expansion [3-10].

Transcriptional dysregulation is a central pathogenic mechanism in HD [11,12]. Gene expression profiling of *in vitro *and *in vivo *models of HD such as the R6/2 mouse model reveals the identities of specifically altered mRNAs that recapitulates those altered in human HD patient brains [13,14]. Indeed, comparison of gene expression studies shows remarkable concordance between mouse models and human HD brain [15]. Furthermore, expression profiling studies suggest that more accessible tissues such as muscle may be suitable for gene expression analyses [16], although the use of blood is more controversial [17,18].

Reverse transcriptase quantitative polymerase chain reaction (qRT-PCR) is a powerful tool to obtain data about gene expression. Normalisation enables one to determine the change in mRNA levels of a gene of interest from multiple samples versus one or more reference gene(s), often a housekeeping gene [19-21]. Reference genes are selected on the basis of constitutive expression across samples to allow the reliable quantification of changes in gene expression [21]. At present, a universally optimal reference gene has not been identified for any organism that can be used across different tissue types or disorders.

Clearly, in a disease such as HD where transcriptional dysregulation is a prominent pathological feature, identification of suitable reference genes throughout the disease time-course is critical. This is amply demonstrated by data showing that beta-actin (*Actb*), a commonly-used reference, is dysregulated at late time points in R6/2 mouse brain [22]. However, a systematic study comparing the suitability of different candidate reference genes in HD has not been performed. To remedy this situation, 12 housekeeping genes were examined to determine their utility as references in three distinct dissected brain regions from 15 week old R6/2 mice and littermate controls. We found that the expression levels of candidate reference genes varied according to genotype and brain region, highlighting the necessity of identifying suitable references in the tissue or cell line under study. GeNorm software was used to identify the three most stably expressed genes for each brain region, from which the geometric mean was derived as a reliable normalisation value for relative gene expression level analyses. Taken together, we propose that the method outlined in this study can be applied in different tissue samples in order to reliably generate gene expression changes as a biomarker in genetic and pharmacological approaches to modifying HD related phenotypes.

## Results

### Mice

Hemizygous R6/2 transgenic mice were bred and reared in our colony by backcrossing R6/2 males to (CBA × C57Bl/6) F1 females (B6CBAF1/OlaHsd, Harlan Olac, Bicester, UK) [23]. The R6/2 mouse model of HD ubiquitously expresses exon 1 of the human HD gene with over 150 CAG repeats, and develops neuropathology, cognitive and motor symptoms. R6/2 mice were always housed with wild type mice and were subject to a 12-h light: 12-h dark cycle. All animals had unlimited access to water and breeding chow and housing conditions and environmental enrichment were as previously described [24]. R6/2 transgenic mice and wild-type littermate controls were sacrificed by cervical dislocation and brains rapidly removed. Striatum, cortex and cerebellum were dissected and flash-frozen in liquid nitrogen and stored at -80°C until use. The guidelines for animal care and use were in accordance with Home Office regulations.

### RNA extraction and reverse transcription

Quantitative RT-PCR (qRT-PCR) represents a powerful tool for detection and quantification of mRNA with high sensitivity, good reproducibility and a wide dynamic range. The principles of qRT-PCR: RNA extraction, reverse transcription (RT), quantitative PCR, fluorescent chemistry and appropriate real-time platforms are all extensively described in the literature [20,25], and through internet forums . Each step can directly impact the reliability of the assay and quality assessment is essential to minimise variability (see Additional File 1).

To optimise RNA extraction from dissected brain regions, we use QIAZOL together with RNeasy Mini kits according to the manufacturers protocol (Qiagen), eluting into either 50 μl of RNase-free water for striatal RNA or 100 μl for cerebellar or cortical RNA. We routinely incorporate DNase treatment as part of the RNeasy procedure (Qiagen) to remove any residual DNA, and perform "minus-RT" controls as well as no-template controls in the PCR analyses. In order to improve signal-to-noise ratio, we use dissected brain regions such as striatum, cortex or cerebellum. RNA quality encompasses both purity and integrity, and we use the Agilent Bioanalyser 2100 RNA Nano assay to assess this via the 28S/18S rRNA ratio, and to determine the RNA concentration (see Additional File 1). Valid alternatives include the nanodrop procedure or the use of 3':5' assays [25].

The RT step is the source of most of the variability and is critically dependent on both enzyme choice and priming method. Target gene-specific primers can be used successfully to eliminate spurious transcripts but then separate RT reactions are needed for each gene of interest, which is inefficient when surveying several genes, and furthermore, different reactions do not have the same cDNA synthesis efficiency. We therefore use MMLV RT-ase (Invitrogen) together with random hexamers to synthesise a cDNA pool which is used for a number of different target-specific qPCR assays (see Additional File 1). In order to minimise variability, we prepare all experimental samples intended for comparison by qRT-PCR in parallel, adding the same amount of RNA template to each RT reaction. Reverse transcription of total RNA (either 5 μg of RNA from cerebellum and cortex or 1 μg of striatal RNA) was performed with MMLV RTase (Invitrogen). The cDNA samples were then cooled to 4°C and diluted 1:10 before storing at -20°C.

### Real-time quantitative PCR (qRT-PCR)

QPCR uses fluorescent reporter dyes to combine the amplification and detection steps of the PCR reaction. The qPCR assay relies on measuring the increase in fluorescent signal, which is proportional to the amount of DNA produced during the logarithmic phase of the PCR cycle. Individual reactions are characterised by the PCR cycle at which fluorescence first rises above a defined or threshold background fluorescence, a parameter known as the threshold cycle (C_t_). The more target there is in the starting material, the lower the C_t_. This correlation between fluorescence and amount of amplified product permits accurate quantification over a wide dynamic range, while retaining the sensitivity and specificity inherent in a conventional PCR. An additional benefit is conferred by reducing variability which can be introduced post-RT-PCR (such as ethidium bromide gel staining and densitometric analysis). We use two methods for the quantitative detection of the amplicon: (a) gene-specific fluorescent Taqman probes, or (b) SYBR green, a non-sequence specific fluorescent intercalating double-stranded DNA binding dye. Both types of reaction can work extremely well, with comparable dynamic range and sensitivity. An additional advantage conferred by the Taqman system is the use of probes labelled with different reporter dyes, allowing the detection and quantification of multiple target genes in a single (multiplex) reaction.

qPCR reactions are performed in triplicate with 5 μl of diluted cDNA template in a 25 μl reaction containing Precision Mastermix (PrimerDesign), 300 nM primers and 200 nM probe using the Opticon 2 and Chromo4 real-time PCR machines (Biorad). We preferentially use the Taqman system and therefore, primers and probes were designed using Primer Express software (Table 1). Probes were tagged with a FAM fluophore and a TAMRA quencher. Crossing threshold data were obtained during the logarithmic phase of amplification where efficiency was close to 100%.

**Table 1:** Primer and probe lists.

**GENE**	**FORWARD PRIMER**	**REVERSE PRIMER**	**PROBE (5' FAM, 3' TAMRA)**
Arl6ip2	TTTGGAATGAAGTGTTTGTGATTGA	GGCACCCTGGGTATCCATTA	AGACCTAATGGAACAAAAGTGGCTGTGCTG

Actb	GCTTCTTTGCAGCTCCTTCGT	CCAGCGCAGCGATATCG	CGGTCCACACCCGCCACCAG

Bdnf I	GCAAAGCCGAACTTCTCACAT	GCAACCGAAGTATGAAATAACCATAG	TTCCACCAGGTGAGAAGAGTGATGACCAT

Bdnf IIa	ACAGAGCCAGCGGATTTGTC	GCAACCGAAGTATGAAATAACCATAG	TTCCACCAGGTGAGAAGAGTGATGACCAT

Bdnf IIb	AGTTGGCTTCCTAGCGGTGTAG	GCAACCGAAGTATGAAATAACCATAG	TTCCACCAGGTGAGAAGAGTGATGACCAT

Bdnf IIc	TGCAACTCTTTATCACCAGGATCTA	GCAACCGAAGTATGAAATAACCATAG	TTCCACCAGGTGAGAAGAGTGATGACCAT

Bdnf III	GGGCCGGATGCTTCCTT	GCAACCGAAGTATGAAATAACCATAG	TTCCACCAGGTGAGAAGAGTGATGACCAT

Bdnf IV	CTGCCTTGATGTTTACTTTGACAAG	GCAACCGAAGTATGAAATAACCATAG	TTCCACCAGGTGAGAAGAGTGATGACCAT

Bdnf V	GGGATCCGAGAGCTTTGTG	GCAACCGAAGTATGAAATAACCATAG	TTCCACCAGGTGAGAAGAGTGATGACCAT

Bdnf VI	TCCTGAGGAAGTGAAAGTTTTGACT	GCAACCGAAGTATGAAATAACCATAG	TTCCACCAGGTGAGAAGAGTGATGACCAT

Bdnf VII	GATTGCTGAAAATGGTGTCGTAAA	GCAACCGAAGTATGAAATAACCATAG	TTCCACCAGGTGAGAAGAGTGATGACCAT

Bdnf VIII	CTGGATGCCGCAAACATGTC	CTGCCGCTGTGACCCACTC	TCACACACGCTCAGCTCCCCACGG

Cnr1	CACAAGCACGCCAATAACACA	ACAGTGCTCTTGATGCAGCTTTC	CCAGCATGCACAGGGCCGC

Darpp32	CCCGACAGGTGGAGATGATC	GCTGCACAGCTTTCAGTGATG	CTGCCATGCTTTTCCGGGTCTCAGA

Drd2	ACACCACTCAAGGGCAACTGT	GGCGGGCAGCATCCA	CCCTGAGGACATGAAACTCTGCACCG

Grin1	TCAGTGTGTGAGGACCTCATCTCT	GAGTGAAGTGGTCGTTGGGAGTA	CAGGTCTACGCTATCCTAGTTAGTCACCCGCC

Hdh	CTCAGAAGTGCAGGCCTTACCT	GATTCCTCCGGTCTTTTGCTT	TGAATCTTCTTCCATGCCTGACCCGA

*HD *(transgenic)	GCTGCACCGACCGTGAGT	CGCAGGCTGCAGGGTTAC	CAGCTCCCTGTCCCGGCGG

Igfbp5	AAGGATTCTACAAGAGAAAGCAGTGTAA	ACTTGTCCACACACCAGCAGAT	TCCCGTGGCCGCAAACGTG

Kcnk2	GACTACGTGGCAGGTGGATCA	GCCAGCCCAACGAGGAT	AATATCTGGACTTCTACAAGCCTGTGGTGTGGT

Nab2	GGGAGGGCAAACAGCTTAGC	AGTGTTGTCCCTCATGCAGAACT	ACCATCAACGAGGCTGCTGCC

Nr4a2	ATTTCCTCGAAAACTCCAATAACTCT	TGAGGCGAGGACCCATACTG	CTGAAGCCATGCCTTGTGTTCAGGC

Pcp4	CTGAGCTGTTCTGTGGGACCTA	CGCTCCGGCACTTTGTCT	CTGCGGAGTCAGGCCAACATGA

Penk1	ATGCAGCTACCGCCTGGTT	GCAGCTGTCCTTCACATTCCA	AGGCGACATCAATTTCCTGGCGTG

Polr2a	GGTGCTGAGTGAGAAGGATGTAGA	ATGCCCAGTACCGTGAAGATCT	TGCGCACCACGTCCAATGATATTGTG

Psme1	TGATGACCAACCTTCACACCAA	TCACCCCTCTCGGAGAAGTACT'	CTGGAAGGCTTCCACACGCAGATCTCC

Sez6	TGTGCCAGTGGGACCTAAGC	TCACAGACATATTGCACAGTTGCT	CATGCCAGAGAGTGACATCTTGCCA

Tbr2	CAAAGGCTTCCGGGACAAC	GGGAGATGGAGTTAACCTGTCATTT	CGATTCCATGTACACGGCTTCAG

UchL1	GGTACCATCGGGTTGATCCA	AACTGTTTCAGGACGGATCCA	AACCAAGACAAGCTGGAATTTGAGGA

Wdr6	GAACAAGCACAAGATGATCAAGGT	GCCTATCGTTGTCAAGCTCACA	TGAGACCAGGTACATGTCTCTTGCTATT

The forward and reverse primer sequences, together with the Taqman probe sequences for each gene routinely used in our laboratory are listed.

**KEY: Arl6ip2 **(ADP-ribosylation factor-like 6 interacting protein 2); **Actb **(beta-actin); **Bdnf **(Brain-derived neurotrophic factor; Roman numerals refer to promoter-specific amplification); **Cnr1 **(Cannabinoid receptor 1); **Darpp32 **(Dopamine and cAMP regulated neuronal phosphoprotein, also known as Ppp1r1b, protein phosphatase 1, regulatory subunit 1B); **Drd2 **(Dopamine D2 receptor); **Grin1 **(glutamate receptor, ionotropic, NMDA1 {zeta 1}); **Hdh **(Murine huntington's disease gene homologue); **Htt **(Human Huntington's disease gene, used to detect human exon 1 transgene); **Igfbp5 **(insulin-like growth factor binding protein 5 precursor); **Kcnk2 **(potassium channel, subfamily K, member 2); **Nab2 **(Ngfi-A binding protein 2); **Nr4a2 **(nuclear receptor subfamily 4, group A, member 2); **Pcp4 **(Purkinje cell protein 4); **Penk1 **(Preproenkephalin); **Polr2a **(RNA polymerase II {DNA directed} polypeptide A); **Psme1 **(proteasome activator 28-α subunit); **Sez6 **(seizure related gene 6); **Tbr2 **(eomesodermin homolog), **Uchl1 **(ubiquitin carboxyl-terminal hydrolase L1), **Wdr6 **(WD repeat domain 6).

### Quantification strategy

Finally, the quantification strategy must be considered. Absolute quantification correlates the PCR signal to input copy number using a calibration curve, and is dependent on equivalent amplification efficiencies for both the native target and the calibration curve. Absolute quantification should be performed in situations where it is necessary to determine the transcript copy number, such as determining the titre of virus particles in blood. In contrast, relative quantification measures the relative change in mRNA expression levels and is easier to perform than absolute quantification because a calibration curve is not necessary. Relative quantification is based on the expression levels of target gene(s) versus reference gene(s) and the relative quantities can be compared across multiple experiments. We use relative quantification when considering genetic and/or pharmacological modulation of HD related phenotypes. With respect to understanding the effect of a manipulation in a pathogenic pathway, it is more informative to state that a given treatment increases the expression of gene *A *by 5 fold, than if we state that the treatment increases the expression of gene *A *from 1000 copies to 5000 copies per cell [19]. Various mathematical models have been established to calculate the expression of a target gene in relation to an adequate reference, based on the comparison of the distinct cycle threshold values (C_t_) at a constant level of fluorescent with, or without PCR efficiency correction. We use a well-characterised 2^-ΔΔCt ^equation, as our target and reference PCR amplicons are generated with equivalent efficiencies [20].

### Data analysis using the 2^-ΔΔCt ^method

The C_t _values provided from real-time PCR instrumentation are easily imported into a spreadsheet programme such as Microsoft Excel. When designing the experiment, we aim for an n ≥ 6 per group and whenever possible, reactions are performed in triplicate for both samples and controls. During 2^-ΔΔCt ^analysis, each sample is treated separately (see Additional File 2). We first ensure that the data from each triplicate fall within 1 C_t _and remove clear outliers (> 2 standard deviations) from the analysis. We determine the mean C_t _and standard deviation (SD) from the triplicates and use the means of each sample for analysis. The first step of analysis (ΔC_t_) is to normalise all the samples with respect to the least expressed sample, thus subtracting the highest C_t _value from the C_t _value of each sample. In this step, the least expressed sample will have a ΔC_t _value of 0 and all the other samples will have negative values (see Additional File 2). The second step is to perform the function (ΔC_t sample _- ΔC_t reference_), which gives a ΔΔC_t _value. The third step is to calculate 2^-ΔΔCt^, which yields the expression ratio (as a positive integer) for each individual sample. After performing the 2^-ΔΔCt ^calculation for each sample, we calculate the means for each group in addition to determining the standard deviation and standard error of the mean (SEM) thus providing a relative expression ratio (in arbitrary units) and variation between ratios (see Additional File 2). The expression ratio data are amenable to statistical tests.

### Dysregulation of commonly-used normalisation genes in aged R6/2 mice

**Figure 1:**
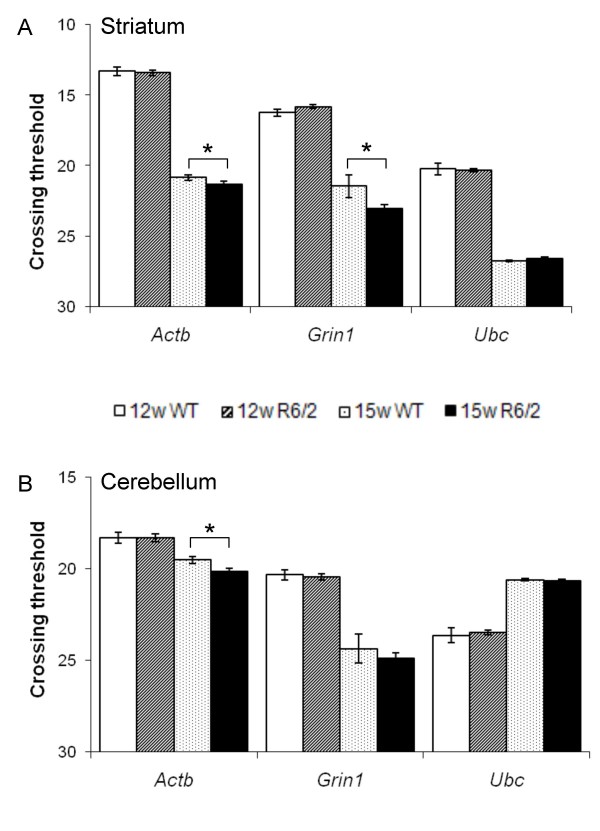
**Progressive gene dysregulation in R6/2 mice over time**. (A) Raw crossing threshold data for *Actb*, *Grin1 *and *Ubc *in 12 and 15 week old R6/2 transgenic mice and wild-type littermates in striatum shows an increase in crossing threshold data for both *Actb *and *Grin1 *but not *Ubc*, despite carefully controlling for RNA amounts. (B) Similar data as for (A), except that RNA extracts are from cerebellum. 12 week old wild-type (WT) = white bars, 12 week old R6/2 mice = stripes, 15 week old WT = dots, 15 week old R6/2 = solid fill bars. Error bars are S.E.M. (n = 10), * p < 0.05.

Modulation of the R6/2 phenotype by genetic or pharmacological methods involves a comprehensive battery of behavioural tests which are performed up to 14 or 15 weeks of age [24]. At the end of the trial, mice are sacrificed and tissues are taken for further biochemical analyses such as aggregate quantification [26], or gene expression analyses. However, a systematic study to identify the most suitable reference genes for use in HD mouse models has not been performed. The majority of researchers in the HD field appear to use beta-actin (*Actb*) and the NMDA receptor subunit 1 (*Grin1*) as reference genes. It was recently reported that both *Actb *and *Grin1 *were down-regulated at 14 weeks of age in R6/2 brain regions [22]. We therefore set out to determine the suitability of *Actb *and *Grin1*, together with ubiquitin C (*Ubc*) at 12 and 15 weeks of age in striatum and cerebellum of transgenic R6/2 mice and matched controls. We found down-regulation of both *Actb *and *Grin1 *mRNA levels in both striatum (Figure 1A, p < 0.05) and cerebellum (Figure 1B, p < 0.05) of 15 week R6/2 mice despite equivalent expression levels in transgenic and control mice at 12 weeks of age. Therefore, the suitability of *Actb *and *Grin1 *as references became questionable.

### Identification of suitable reference genes in dissected brain regions

Given the progressive nature of the transcriptional dysregulation phenotype in the R6/2 mice, with down-regulation of commonly used reference genes such as *Actb*, it was critical to determine which genes were most suitable for normalisation at later time points. However, to validate the stable expression of a given control gene, prior knowledge of a reliable measure to normalise this gene is required. In order to circumvent this circular problem, we took advantage of the principle that the expression ratio of two ideal internal control genes will be near-identical in all samples tested, regardless of the experimental condition [21]. We therefore utilized the geNorm Housekeeping Gene Selection Kit (PrimerDesign) to evaluate 12 commonly used housekeeping genes [21] in 3 different dissected brain regions from 15 week old wild-type and R6/2 mice. Reference genes tested were 18S (18S ribosomal RNA subunit), *Actb *(beta-actin), *Atp5b *(ATP synthase subunit 5b), *B2m *(beta-2 microglobulin), *Canx *(calnexin), *Cyc1 *(cyclin D1), *Eif4a2 *(eukaryotic initiation factor 4a2), *Gapdh *(glyceraldehyde-3-phosphate dehydrogenase), *Rpl13a *(ribosomal protein L13a), *Sdha *(succinate dehydrogenase complex, subunit A), *Ubc *(ubiquitin C) and *Yhwaz *(phospholipase A2).

**Figure 2:**
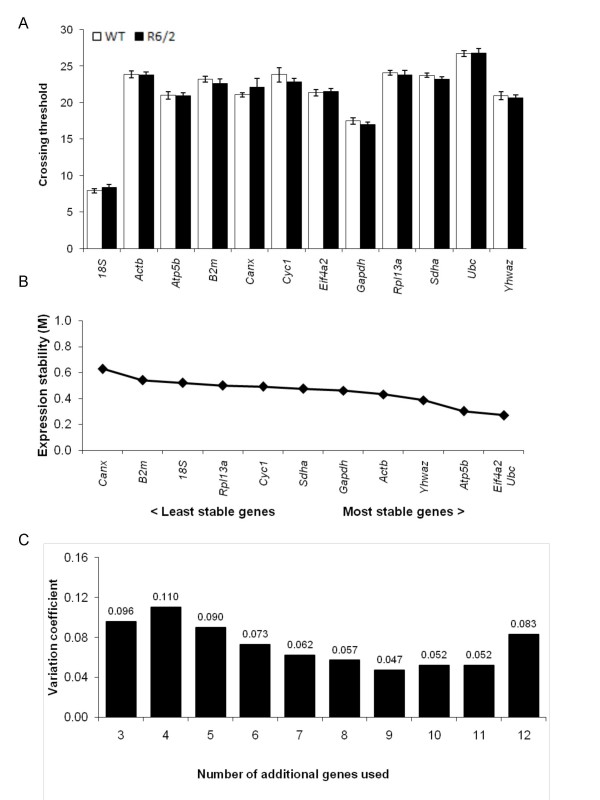
**GeNorm analyses to identify optimal reference genes in the striatum**. (A) Raw crossing threshold (C_t_) data for a panel of 12 potential references from the geNorm kit in wild-type (open bars) and R6/2 (filled bars) mice. (B) Raw C_t _data was subjected to analysis with the geNorm applet which automatically calculates the gene-stability measure *M*, which is an average pairwise variation of a particular gene with all other control genes. Therefore, genes with the lowest *M *value have the most stable expression, in this case across genotypes (i.e. comparing wild-type and R6/2 mice). Expression stability is plotted for each of the potential reference genes, progressing from the least stable genes with a higher *M *value to the most stable genes with a lower *M *value. (C) In order to measure expression levels accurately, normalization by multiple housekeeping genes is optimal. The graph illustrates the levels of variation in average reference gene stability with the sequential addition of each reference gene to the equation, starting with the three most stably expressed genes on the left with the inclusion of a 4^th ^gene and so on, moving to the right. This measure is known as pairwise variation (*V*), the values of which are indicated above each bar, with a score of <0.15 as a target.

Equal amounts of RNA extracted from striatum dissected from 9 R6/2 mice and 9 wild-type controls were reverse-transcribed and used as a template for real-time PCR using the primer/probe sets provided according to the manufacturers protocol (PrimerDesign). Each reaction was performed in triplicate and the C_t _values for each sample were averaged to obtain raw C_t _values (Figure 2a). The raw C_t _values were transformed into relative quantification data using the 2^-ΔΔCt ^method and used to prepare an input file for geNorm analysis. The geNorm output ranks the candidate reference genes according to their expression stability (Figure 2b) and determines the number of reference genes required for optimal normalisation, expressed as pairwise variation (V) (Figure 2c). We identified *Ubc *(ubiquitin C), *Eif4a2 *(eukaryotic initiation factor 4a2) and *Atp5b *(ATP synthase subunit 5b) as being the most stably expressed genes in 15 week wild-type and R6/2 mouse striatum (Table 2). To increase confidence in our data, we confirmed our findings in a separate cohort of samples (data not shown).

**Table 2:** Control genes ranked in order of their expression stability.

**Striatum**	**Cerebellum**	**Cortex**
UbC	Canx	Atp5b
Eif4a2	Atp5b	Rpl13a
Atp5b	Eif4a2	Canx
Yhwaz	18S	UbC
Actb	Rpl13a	18S
Gapdh	Cyc1	Eif4a2
Sdha	UbC	Yhwaz
Cyc1	Sdha	Cyc1
Rpl13a	Actb	Sdha
18S	B2M	Gapdh
B2M	Gapdh	B2M
Canx	Yhwaz	Actb

Housekeeping genes are ranked in order of their expression stability, and therefore their suitability for use as a reference for the striatum, cerebellum and cortex. The most suitable genes are listed first.

Brain tissue is not homogenous and is composed of widely differing cell types. Consistent with this, microarray analysis of distinct brain regions and cell types shows highly specific transcriptional profiles [27-31]. We therefore set out to determine whether the optimal reference genes identified for use in striatum were also suitable for use as references in two other brain regions, the cerebellum and the cerebral cortex. GeNorm analysis was performed using RNA prepared from dissected cerebellum (see Additional File 3) and cortex (see Additional File 4) in precisely the same manner as for the striatum. We found that the tissue source determined the choice of genes for normalisation, with *Canx *(calnexin), *Atp5b *and *Eif4a2 *identified for normalisation in the cerebellum; and *Atp5b*, *Rpl13a *(ribosomal protein L13a) and *Canx *being suitable choices for the cortex. The cortex showed more expression stability variation than either the striatum or cerebellum, which may reflect a greater heterogeneity in cell types and function.

### Validation of reference genes identified through geNorm analyses

The geNorm analyses identified the most stably expressed tissue-specific reference genes from our initial panel of twelve candidates. We concluded that in order to measure expression levels of our genes of interest accurately by relative quantification, normalization by multiple housekeeping genes would be more robust. We therefore chose to use the geometric mean of at least the best three reference genes identified for each tissue in our analyses as it indicates the central tendency, or typical value of a set of numbers [21]. Furthermore, the geometric mean is more appropriate than the arithmetic mean as we would expect that changes in the gene expression would occur in a relative fashion and hence we can control better for possible outlying values and abundance differences between the different genes. In order to establish relative expression analyses for dysregulated genes in the striatum of R6/2 mice, we first confirmed that the dynamic C_t _range of the reference genes was equivalent to the majority of our genes of interest and that the amplification efficiencies of the reference genes and genes of interest were equivalent.

**Figure 3:**
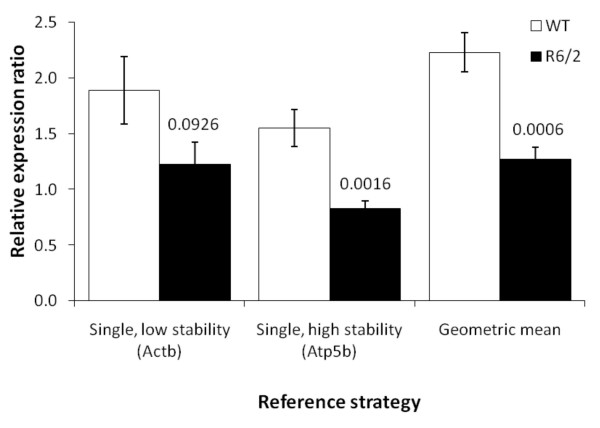
**Comparison of normalisation strategy**. Relative expression analyses using either a single reference gene with low stability between wild-type (WT, open bars) and R6/2 (filled bars) cortex such as *Actb*, a single reference gene with high stability between the two genotypes such as *calnexin *or the geometric mean of three highly stable reference genes. P-values above the R6/2 bars show that while a single, stably expressed gene (p = 0.0016) is a better calibrator than a gene with low expression stability between the two genotypes (p = 0.0926), calculating the geometric mean of three stably expressed genes is a far superior normalisation strategy (p = 0.0006). Error bars are S.E.M (n = 6).

In order to test the validity of using the geometric mean of three reference genes, we focused on expression of *Bdnf *in the cortex as this is a target gene of interest in HD [32-36]. We compared the relative expression analysis output for the coding exon of *Bdnf *from 12 week R6/2 and matched littermate controls with either a single reference gene (with low or high stability between the two genotypes) or with the geometric mean of three reference genes in wild-type and R6/2 cortex. In the first instance, we found that using a single reference gene with a high stability value between the two genotypes (such as *Canx *in the cortex; p = 0.0016) is superior to using a single reference gene with a poor stability profile between the two genotypes (such as *Actb*, p = 0.0926) (Figure 3). Furthermore, it was clear that using the geometric mean of three reference genes further decreased the p-value and therefore increased the sensitivity and reliability of detecting the difference in *Bdnf *expression compared to using a single reference gene (p = 0.0006) (Figure 3). Therefore, using the geometric mean of three reference genes markedly improves our ability to detect subtle effects on *Bdnf *expression by an experimental modulation.

### BDNF expression analyses

**Figure 4:**
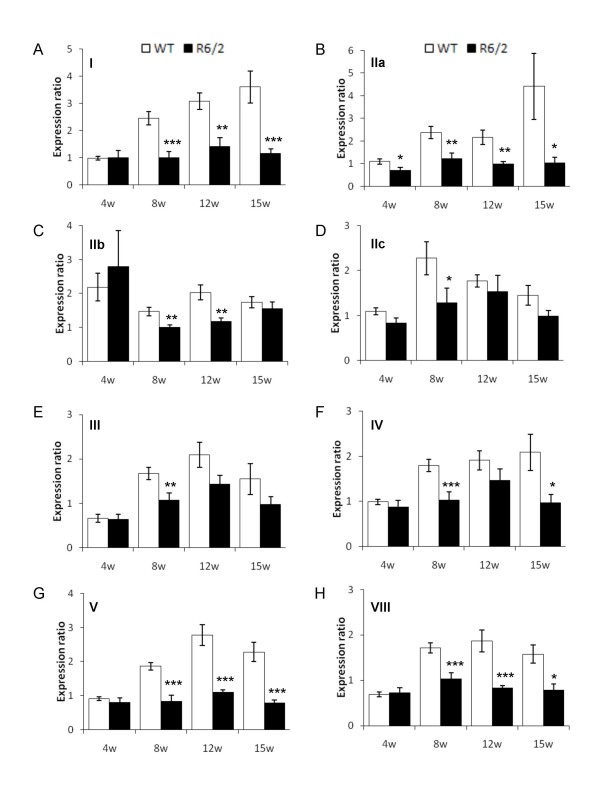
***Bdnf *promoter-specific expression analyses in R6/2 cortex**. Relative expression ratios to the geometric mean of three housekeeping genes for *Bdnf *promoter-specific transcripts in R6/2 (filled bars) and littermate wild-type controls (open bars). Data from 4, 8, 12 and 15 weeks (4 w, 8 w, 12 w and 15 w) represents the pathogenic time course from pre-symptomatic stages through to late symptomatic stages for *Bdnf *promoter-specific transcripts (A) I, (B) IIa, (C) IIb, (D) IIc, (E) III, (F) IV, (G) V and (H) coding exon VIII, according to Liu et al., 2006 [39]. Error bars are S.E.M (n = 8), * p < 0.05, ** p < 0.01, *** p < 0.001.

**Figure 5:**
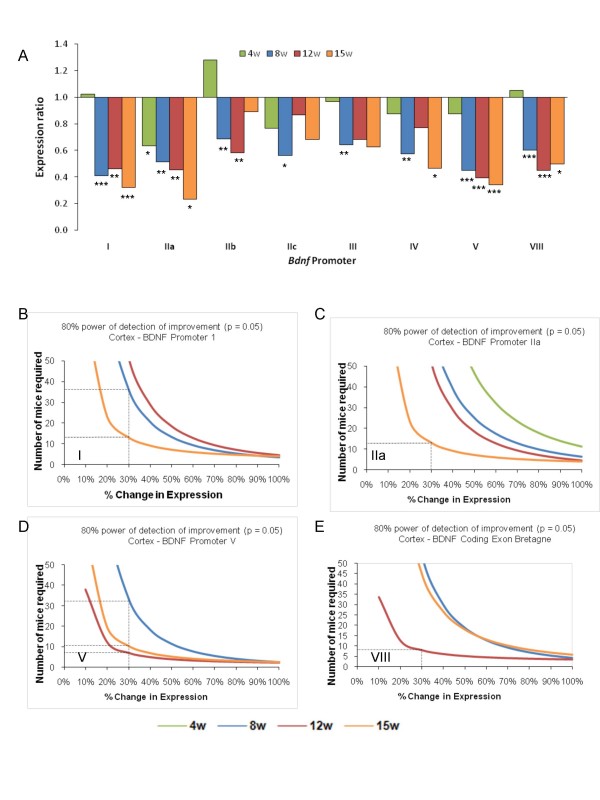
**Power calculations determine the optimal *Bdnf *assays for use as preclnical assessment tools**. (A) Shows the relative expression level of each *Bdnf *promoter-specific transcript expressed as a ratio of R6/2: wild-type from a pre-symptomatic time point (4 weeks, 4 w) to early symptomatic (8 w) and late symptomatic time points (12 and 15 w). Therefore, each bar represents the relative amount of down-regulation of each transcript in R6/2 mouse cortex compared to wild-type. Significant differences between R6/2 and wild-type mice for a specific transcript at the ages indicated are represented with asterisks below the relevant bar. * p < 0.05, ** p < 0.01, *** p < 0.001. Promoter-specific transcript nomenclature is according to Liu et al., 2006 [39]. We performed power calculations (shown is 80% power of detection of improvement at p > 0.05) as previously described [40] for BDNF promoters, with promoters I (B), IIa (C), V (D) and VIII (E) being the best powered to detect potential modulation of *Bdnf *expression levels by genetic or pharmacological approaches (data not shown for remaining promoters). The dotted line illustrates the number of mice needed in order to be able to detect a 30% improvement with 80% confidence.

Brain-derived neurotrophic factor (BDNF) is an important neurotrophic factor involved in regulating neuronal transmission, striatal neuronal survival, and has previously been implicated in HD pathogenesis [37,38]. BDNF mRNA and protein levels are severely decreased in the cortices of numerous HD murine models, including R6/2 mice, and in human HD patients. Assaying BDNF levels therefore holds promise as a useful pre-clinical trial biomarker. Rodent *Bdnf *gene structure and regulation is complicated, with six upstream transcription start sites spliced (either bipartite or tripartite) to a major coding exon [39]. We therefore established Taqman assays utilising a common reverse primer and probe in the coding exon in order to determine the extent of exon-specific *Bdnf *mRNA down-regulation over time in R6/2 mice. We additionally surveyed coding exon-specific *Bdnf *expression. RNA was extracted from cortices of 4, 8, 12 and 15 week old R6/2 transgenic mice and littermate controls and used to synthesize cDNA as a template for qRT-PCR. The geometric mean of C_t _data from three housekeeping genes was used to normalise levels of *Bdnf *transcript using the 2^ΔΔCt ^formula. We observed expression of all *Bdnf *transcripts with the exception of 6a and 6b in R6/2 cortex at all times tested (Figure 4). At 4 weeks of age, there was no difference in *Bdnf *expression level between R6/2 and controls, with the exception of a decrease in promoter IIa-specific transcript in R6/2 mice. However, we saw a significant decline in *Bdnf *expression levels at symptomatic time points from promoters I, IIa, IIb, IV, V, and also in the coding exon VIII. We subjected the data to power calculations as previously described [40] (Figure 5), which can be used in several ways: (1) to determine how many animals would be required to have a reasonable (e.g. > 80%, Figure 5B–E and data not shown) chance of detecting an improvement of a given magnitude at a given *P *value (p < 0.05); (2) to determine the size of an effect one could expect to detect using a given number of animals; (3) to assess the probability of detecting an effect of given magnitude using a given number of animals. Power calculations suggested that transcript-specific analyses from *Bdnf *promoters could be informative when determining the effect of a pre-clinical modulation. For example, 10 samples would be sufficient to observe a 23% and 30% improvement in B*dnf *promoter V expression at 12 and 15 weeks of age respectively with 80% power at p < 0.05 (Figure 5D). However, we obtained less powered to detect improvements in transcription from *Bdnf *promoters I, IIa and VIII in this dataset with statistical confidence (Figures 5B, C and 5E respectively). In order to illustrate this, the dotted lines plotted on Figure 5B–E show the number of mice required to detect 30% improvement in Bdnf transcript expression. We would need 14 mice for *Bdnf *promoter IIa at 15 weeks, 36 mice at 8 weeks, but over 50 mice at 12 weeks; 14 mice for *Bdnf *promoter IIb at 15 weeks but over 50 mice at 12 weeks; 11 mice for *Bdnf *promoter V at 15 weeks but 8 mice at 12 weeks and 34 mice at 8 weeks; 9 mice for *Bdnf *promoter VIII at 12 weeks but over 50 mice at 15 weeks. Therefore, the age at which the levels of a particular transcript are analysed could be important and needs to be surveyed. Obviously, the larger the change in R6/2 *Bdnf *transcript levels with respect to wild type, the smaller the number of samples required to obtain statistical significance.

## Discussion

Recent advances in gene quantification strategies enable the rapid and precise quantification of RNA transcripts. Normalisation is required to remove sampling differences (such as RNA quantity and quality) in order to identify real gene-specific variation. Accurate normalisation of gene expression levels is therefore a prerequisite, especially when studying the biological significance of subtle gene expression differences. The majority of laboratories use a single control gene for normalisation purposes in RT-PCR (both "classical" and real-time RT-PCR), with *Actb*, *Gapdh *and ribosomal RNA being used in over 90% of cases [41]. However, housekeeping gene expression can vary considerably, with obvious impact on meaningful data interpretation. For example, it has been reported that *B2m *(beta-2-microglobulin, a commonly used reference gene) is variably expressed in neuroblastoma cells, depending on the differentiation state of the tumour cells [42], warranting the validation of a reference gene in each experimental system. Additionally, a study showed that a set of genes, including *Actb *varied in expression by 7 to 23-fold across a panel of 60 cell lines [43,44]. In this regard, identifying suitable control genes for normalisation is even more important when working with complex tissues such as brain regions, or with tissues of different histological origins. Consistent with previously published data [22], we found that a commonly used reference gene, *Actb *is specifically dysregulated at later time-points in R6/2 mouse brain regions. Therefore, the use of *Actb *as an appropriate single control normalisation factor is inappropriate, particularly at later time points. This is troubling, as *Actb *appears to be one of the most commonly used normalisation genes in expression analyses in HD.

Clearly, in a disease such as HD, where transcriptional dysregulation is a central pathogenic mechanism [12], it is critical to identify suitable reference genes throughout the disease time-course. However to date, there has been no systematic study comparing the suitability of different candidate reference genes in HD. We therefore chose to survey a panel of 12 housekeeping genes in an unbiased fashion. We found that the expression levels of candidate reference genes varied according to genotype and brain region, highlighting the necessity of identifying suitable references in the tissue under study. Specifically, the three most stable genes for each brain region tested as identified by geNorm analysis (striatum, cerebellum and cortex) are distinct, with only one gene being common to all three (*Atp5b*). This is consistent with the notion that there are highly specific transcriptional profiles in distinct brain regions and cell types.

It has been shown that a conventional normalisation strategy based on a single reference gene can lead to erroneous normalisation up to 6-fold [21]. We therefore propose that a more stringent normalisation strategy should be incorporated, particularly when studying potentially subtle effects upon gene expression levels of a pharmaceutical agent or a genetic modulation strategy. It has been demonstrated that calculating the geometric mean of three reference genes yields a reliable normalisation value, which can then be used to determine relative expression ratios for genes of interest [21]. We therefore surveyed the effect of three different normalisation strategies. We found a stable reference gene to use as a single normaliser is superior to a gene whose expression is unstable between the experimental conditions. However, calculating the geometric mean of three stably expressed reference genes and subsequently using this normalisation factor in subsequent analyses is preferable. Thus, it is critical not only to identify the most suitable reference genes for expression analyses by an unbiased method in the tissue/cell line of interest, but also to utilise a precise normalising strategy in order to derive meaningful results. This is particularly applicable to studies interrogating the molecular mechanism underlying HD pathogenesis and mechanisms by which a particular intervention may exert a beneficial effect.

*Bdnf *gene transcription is induced by wild-type, but not mutant Htt through interactions with NRSF [32]. In addition, *Bdnf *mRNA and protein levels are severely decreased in the cortices of numerous HD murine models as well as in post-mortem human HD cortex [37]. Interestingly, gene expression profiling of multiple HD mouse models and comparison to human HD brain suggests that *Bdnf *depletion plays a major role in striatal degeneration [15]. Remarkably, of all HD mouse models, transcriptional profiles of *Bdnf *knock-out mice are the most similar to gene expression changes identified in HD human brain, suggesting that decreased *Bdnf *expression is a key pathogenic feature in HD, possibly through reduced corticostriatal BDNF transport [33,38]. Furthermore, BDNF levels influence HD onset and progression and up-regulating *Bdnf *expression in HD mouse models through exercise, environmental enrichment, treatment with compounds or by adenoviral injection have all shown promise in ameliorating HD-related symptoms [33,34,36,45,46]. Therefore, there is precedence for *Bdnf *modulation as a therapeutic approach in HD. Taken together, it is critical that we can reliably measure *Bdnf *levels, either when directly attempting to modulate *Bdnf *expression; or for use as a biomarker in the context of other disease-modifying therapies, particularly those aimed at correcting gene expression deficits, such as histone deacetylase inhibitors [22,47-51]. We have therefore developed and validated Taqman-based real-time assays for promoter-specific *Bdnf *gene expression in the cortex of R6/2 mice. Indeed, in this context, we have expanded the scope of interrogating *Bdnf *expression with respect to HD. *Bdnf *gene structure and regulation is complex, with upstream transcription start sites spliced to a coding exon, yielding distinct pro-*Bdnf *molecules which are cleaved to yield a mature BDNF protein. The complexity of the *Bdnf *gene facilitates precise regulation of BDNF with respect to the diversity of its functions. Previously reported investigations into *Bdnf *gene expression dysregulation were based on the four originally described distinctive promoters in the *Bdnf *gene [32-38]. However, the previous work on *Bdnf*'s gene structure was incomplete, and it has recently been extended with the identification of six upstream transcription start sites spliced (either bipartite or tripartite) to a major coding exon [39] (see Additional File 5). We have capitalised on the more detailed knowledge of *Bdnf *gene structure by designing promoter-specific Taqman assays. We have therefore not only confirmed previously published promoter-specific dysregulation in the R6/2 mouse model of HD, but expanded this knowledge. Of particular interest, we have designed specific assays from each of the variants arising from alternative splicing events through the intra-exonic splice sites in promoter II, which has previously been shown to be down-regulated in the R6/2 striatum. Specifically, we have shown that while all three splice variants from Bdnf promoter II are dysregulated, Bdnf transcript IIa is progressively dysregulated to a further extent and is more statistically significant. Furthermore, power calculations suggest that we can effectively use *Bdnf *expression analyses as an output measure in a pre-clinical trial. Finally, recently published data has suggested that Bdnf expression dysregulation occurs in a cell-autonomous property in HD [52]. Consistent with this, it appears that gene expression dysregulation may be an intrinsic function of mutant Htt protein, although the precise molecular mechanism remains to be determined [53,54].

## Conclusion

Transcriptional dysregulation is a central pathogenic molecular mechanism in HD, with robust, reproducible and early changes in the expression of specific mRNA moieties. Therefore, analysis of gene expression could be valuable as a biomarker to monitor disease progression and the efficacy of therapeutic agents in clinical trials. However, commonly used reference genes and current methods in the HD field are unsuitable for gene expression analysis. We suggest an improvement on previous methods by demonstrating the identification of suitable and reliable reference genes for dissected brain regions of R6/2 mice, which can be applied to other brain regions, tissues or cell lines. In addition, we illustrate a more robust method for analysis, namely using the geometric mean of three reference genes for relative quantification analyses. These improvements are not novel in that they have been previously described and extensively used in other disease fields. However, they are not routinely used within the HD field. We therefore propose that the methods outlined in this study can be applied in different tissues (including biopsies) and cell lines in order to reliably and accurately determine the relative expression level of target genes in these brain regions, thus increasing the potential of gene expression analysis as a biomarker in HD.

## Methods

Detailed methods and statistical analyses are available as a supplementary section (see Additional File 6).

## List of Abbreviations Used

BDNF: brain derived neurotrophic factor; C_t_: crossing threshold; HD: Huntington's disease; PCR: polymerase chain reaction; polyQ: polyglutamine; qRT-PCR: quantitative real-time PCR; qPCR: quantitative PCR; RT-PCR: reverse transcriptase-PCR.

## Competing interests

The R6/2 mice are licensed by King's College London for commercial use.

## Authors' contributions

CLB initiated this study and participated in its design, including performing the geNorm analyses, optimising the real-time protocol and analyses and drafting the manuscript. HF performed assays and power calculations; GPB participated in the design of this study, provided funding and guidance and helped to draft the manuscript. All authors have read and approved the final manuscript.

## Supplementary Material

Additional file 1 **Gene expression analyses work flow**. Flow diagram to illustrate the work flow involved in gene expression analysis, from data generation (including sample preparation and experimental process) through to data analysis. Click here for file

Additional file 2 **Example worksheet illustrating 2^-ΔΔCt ^analysis process**. Shown is an Excel worksheet illustrating the process of relative expression analysis. In the worksheet are numbers in blue which illustrate each step. (A) Crossing threshold data can be imported into Excel from real-time PCR platforms (shown are the *Bdnf *data only for simplicity). (B) Each sample has been run in triplicate and the means of each sample is calculated. Standard deviations should be checked at this point and should be within 1 C_t_. C) The raw crossing threshold (C_t_) data can be used to plot a graph of the means for each genotype. Shown are means (Avg), standard deviations (SD) and standard error of the mean (SEM) for WT (wild-type) and TG (R6/2) mice (n = 7/genotype). The means are used to generate the graphs, and error bars are SEM. (D) The geometric mean (Geo, or GEOMEAN) is calculated using the raw C_t _data for all three reference genes (*Atp5b*, *Canx*, *Rpl13a*) for each sample. (E) The geometric mean is transformed into a ΔC_t _value, thus expressing each sample with respect to the least expressed sample. To do this, the C_t _of the least expressed sample is subtracted from the current sample C_t_, giving a negative value for each sample. The least expressed sample will have a value of zero. (F) The ΔC_t _is calculated for the *Bdnf *data. (G) The ΔΔC_t _is calculated, by performing the function (C_t _sample - C_t _reference), which will give both positive and negative values. (H) To transform the ΔΔCt values into positive integers that represent the expression levels, use the Excel POWER function, entering 2 as the number and -ΔΔC_t _as the power. The negative sign is necessary in this context. (J) The relative expression levels for each sample can then be used to calculate the means (Avg), standard deviation (SD) and standard error of the mean (SEM) for each genotype. These data are used to generate graphs showing expression ratios for target genes. (K) In addition, the expression ratios can be used as a substrate for statistical analyses such as a Student's t-test. Click here for file

Additional file 3 **GeNorm analyses to identify optimal reference genes in the cerebellum**. (A) Raw crossing threshold (C_t_) data for a panel of 12 potential references from the geNorm kit in wild-type (open bars) and R6/2 (filled bars) mice. Reference genes tested were 18S (18S ribosomal RNA subunit), *Actb *(beta-actin), *Atp5b *(ATP synthase subunit 5b), *B2m *(beta-2 microglobulin), *Canx *(calnexin), *Cyc1 *(cyclin D1), *Eif4a2 *(eukaryotic initiation factor 4a2), *Gapdh *(glyceraldehyde-3-phosphate dehydrogenase), *Rpl13a *(ribosomal protein L13a), *Sdha *(succinate dehydrogenase complex, subunit A), *Ubc *(ubiquitin C) and *Yhwaz *(phospholipaase A2). (B) Raw C_t _data was subjected to analysis with the geNorm applet which automatically calculates the gene-stability measure *M*, which is an average pairwise variation of a particular gene with all other control genes. Therefore, genes with the lowest M value have the most stable expression, in this case across genotypes (ie comparing wild-type and R6/2 mice). Expression stability is plotted for each of the potential reference genes, progressing from the least stable genes with a higher *M *value to the most stable genes with a lower *M *value. (C) In order to measure expression levels accurately, normalization by multiple housekeeping genes is optimal. The graph illustrates the levels of variation in average reference gene stability with the sequential addition of each reference gene to the equation, starting with the most stably expressed genes on the left with the inclusion of a 4^th ^gene etc, moving to the right. This measure is known as pairwise variation (V), the values of which are indicated above each bar. A V score of below 0.15 is the target. Click here for file

Additional file 4 **GeNorm analyses to identify optimal reference genes in the cortex**. (A) Raw crossing threshold data (C_t_) for a panel of 12 potential references from the geNorm kit in wild-type (open bars) and R6/2 (filled bars) mice. Reference genes tested were 18S (18S ribosomal RNA subunit), *Actb *(beta-actin), *Atp5b *(ATP synthase subunit 5b), *B2m *(beta-2 microglobulin), *Canx *(calnexin), *Cyc1 *(cyclin D1), *Eif4a2 *(eukaryotic initiation factor 4a2), *Gapdh *(glyceraldehyde-3-phosphate dehydrogenase), *Rpl13a *(ribosomal protein L13a), *Sdha *(succinate dehydrogenase complex, subunit A), *Ubc *(ubiquitin C) and *Yhwaz *(phospholipaase A2). (B) Raw C_t _data was subjected to analysis with the geNorm applet which automatically calculates the gene-stability measure *M*, which is an average pairwise variation of a particular gene with all other control genes. Therefore, genes with the lowest M value have the most stable expression, in this case across genotypes (ie comparing wild-type and R6/2 mice). Expression stability is plotted for each of the potential reference genes, progressing from the least stable genes with a higher *M *value to the most stable genes with a lower *M *value. (C) In order to measure expression levels accurately, normalization by multiple housekeeping genes is optimal. The graph illustrates the levels of variation in average reference gene stability with the sequential addition of each reference gene to the equation, starting with the most stably expressed genes on the left with the inclusion of a 4^th ^gene etc, moving to the right. This measure is known as pairwise variation (V), the values of which are indicated above each bar. A V score of below 0.15 is the target. Click here for file

Additional file 5 ***Bdnf *gene structure**. This schematic of Mus musculus *Bdnf *gene structure (top panel, accession number AY057907) indicates the "classical" four promoters (blue boxes) and coding exon (white box) and the recently described additional promoters (yellow boxes) [39]. Only the coding exon is translated, giving rise to a protein of 289 amino acids. The NRSE site and intra-exonic splice sites on promoter II are also indicated. The bottom panel shows the splice variants arising from the *Bdnf *gene, including the splice variants arising from intra-exonic splice sites A, B and C in promoter II. Click here for file

Additional file 6 **Methods**. Detailed methods Click here for file
