# Supplementary material for: Optimisation of region-specific reference gene selection and relative gene expression analysis methods for pre-clinical trials of Huntington's disease
Source: Mol Neurodegener. 2008 Oct 27;3:17. doi: 10.1186/1750-1326-3-17 (PMC2584034; doi:10.1186/1750-1326-3-17)
Supplement: Additional file 1 — Gene expression analyses work flow. Flow diagram to illustrate the work flow involved in gene expression analysis, from data generation (including sample preparation and experimental process) through to data analysis. [file 1750-1326-3-17-S1.ppt]

## Slide 1
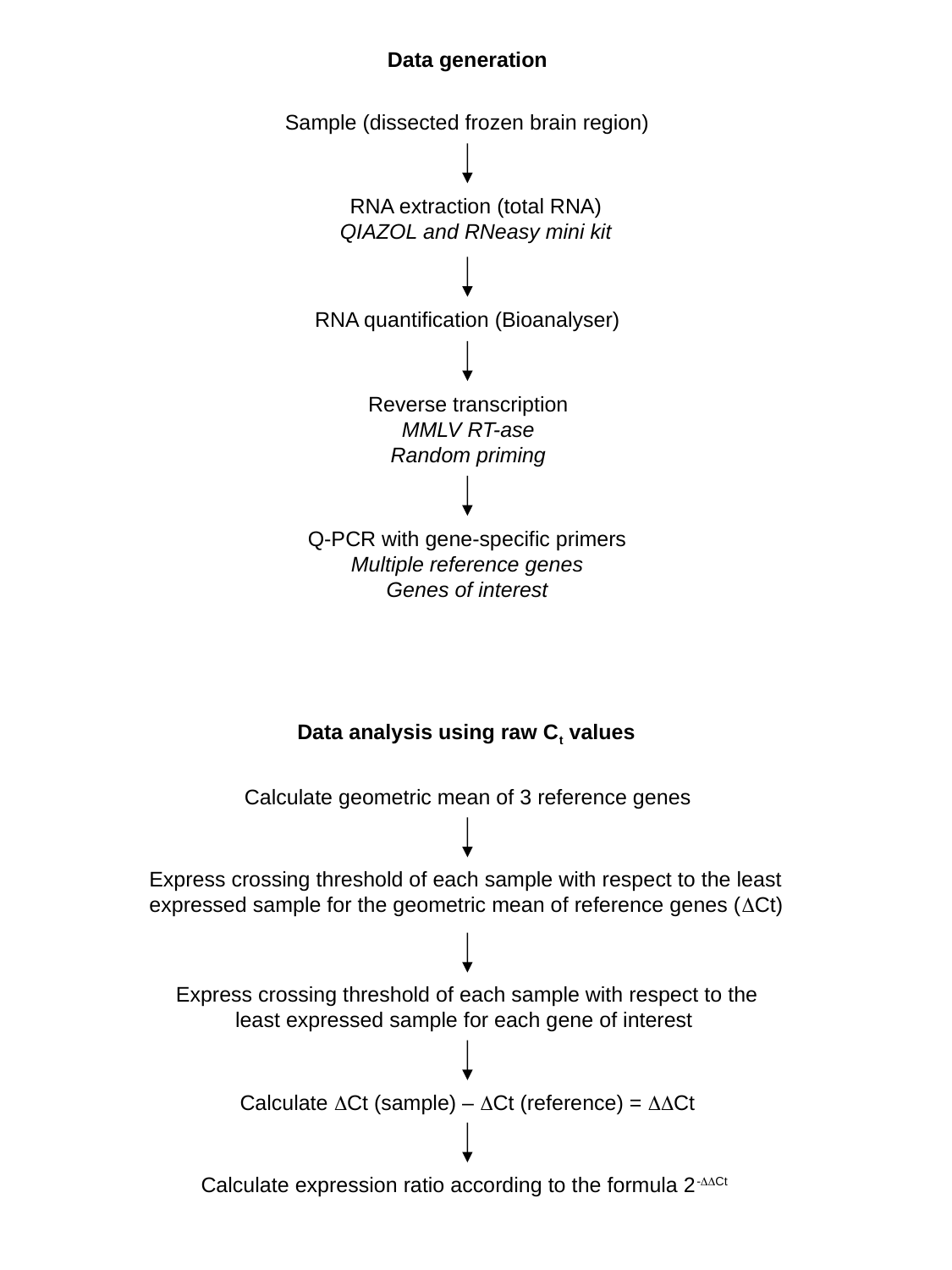

Data generation
Sample (dissected frozen brain region)
RNA extraction (total RNA)
QIAZOL and RNeasy mini kit
RNA quantification (Bioanalyser)
Reverse transcription
MMLV RT-ase
Random priming
Q-PCR with gene-specific primers
Multiple reference genes
Genes of interest
Data analysis using raw Ct values
Calculate geometric mean of 3 reference genes
Express crossing threshold of each sample with respect to the least expressed sample for the geometric mean of reference genes (ΔCt)
Express crossing threshold of each sample with respect to the least expressed sample for each gene of interest
Calculate ΔCt (sample) – ΔCt (reference) = ΔΔCt
Calculate expression ratio according to the formula 2-ΔΔCt
