# Supplementary figures and images for: Optimisation of region-specific reference gene selection and relative gene expression analysis methods for pre-clinical trials of Huntington's disease
Source: Mol Neurodegener. 2008 Oct 27;3:17. doi: 10.1186/1750-1326-3-17 (PMC2584034; doi:10.1186/1750-1326-3-17)

## Slide 1
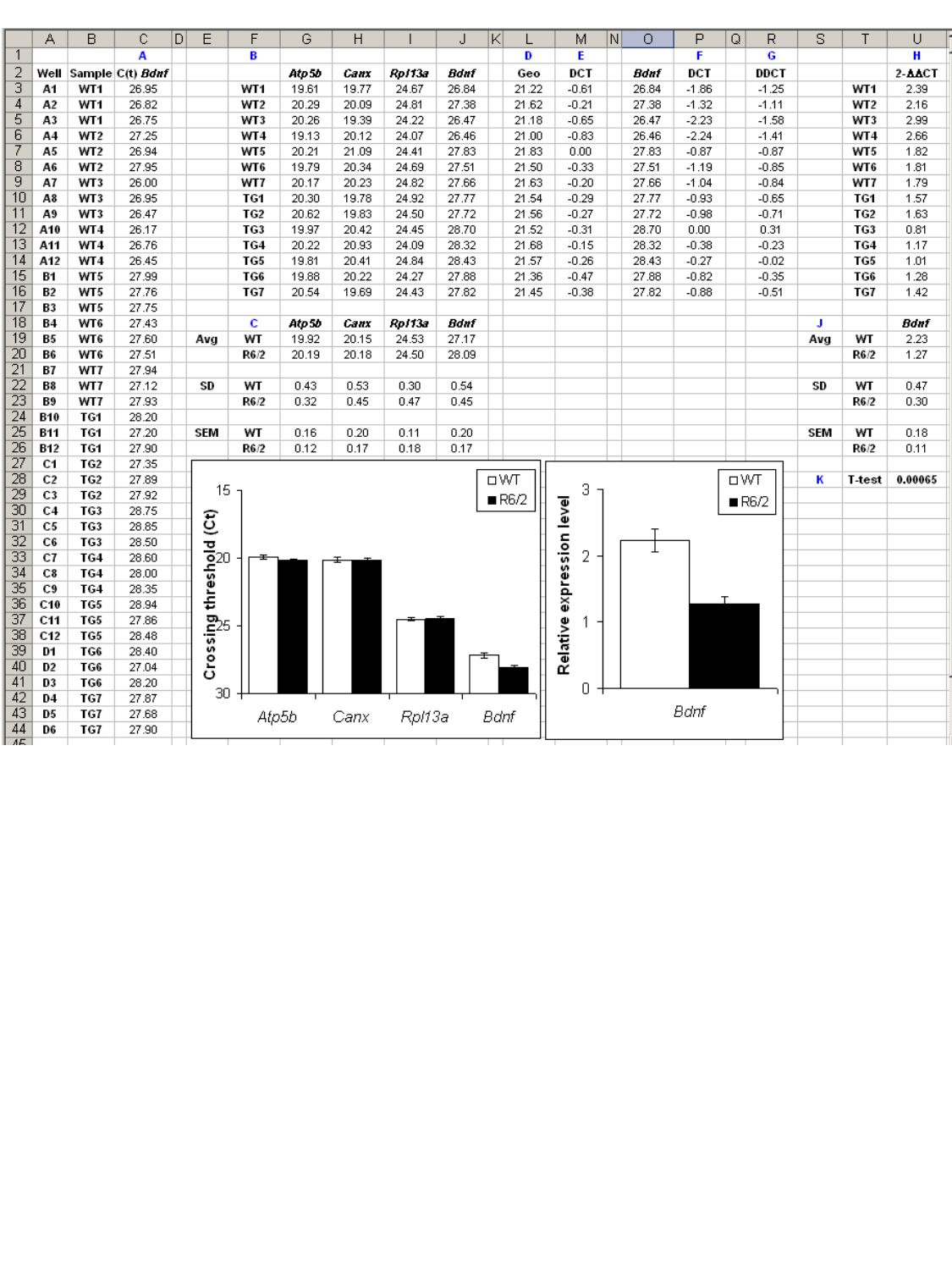

Supplement: Additional file 2 — Example worksheet illustrating 2-ΔΔCt analysis process. Shown is an Excel worksheet illustrating the process of relative expression analysis. In the worksheet are numbers in blue which illustrate each step. (A) Crossing threshold data can be imported into Excel from real-time PCR platforms (shown are the Bdnf data only for simplicity). (B) Each sample has been run in triplicate and the means of each sample is calculated. Standard deviations should be checked at this point and should be within 1 Ct. C) The raw crossing threshold (Ct) data can be used to plot a graph of the means for each genotype. Shown are means (Avg), standard deviations (SD) and standard error of the mean (SEM) for WT (wild-type) and TG (R6/2) mice (n = 7/genotype). The means are used to generate the graphs, and error bars are SEM. (D) The geometric mean (Geo, or GEOMEAN) is calculated using the raw Ct data for all three reference genes (Atp5b, Canx, Rpl13a) for each sample. (E) The geometric mean is transformed into a ΔCt value, thus expressing each sample with respect to the least expressed sample. To do this, the Ct of the least expressed sample is subtracted from the current sample Ct, giving a negative value for each sample. The least expressed sample will have a value of zero. (F) The ΔCt is calculated for the Bdnf data. (G) The ΔΔCt is calculated, by performing the function (Ct sample - Ct reference), which will give both positive and negative values. (H) To transform the ΔΔCt values into positive integers that represent the expression levels, use the Excel POWER function, entering 2 as the number and -ΔΔCt as the power. The negative sign is necessary in this context. (J) The relative expression levels for each sample can then be used to calculate the means (Avg), standard deviation (SD) and standard error of the mean (SEM) for each genotype. These data are used to generate graphs showing expression ratios for target genes. (K) In addition, the expression ratios can be used as a substrate f [file 1750-1326-3-17-S2.ppt]

## Slide 1
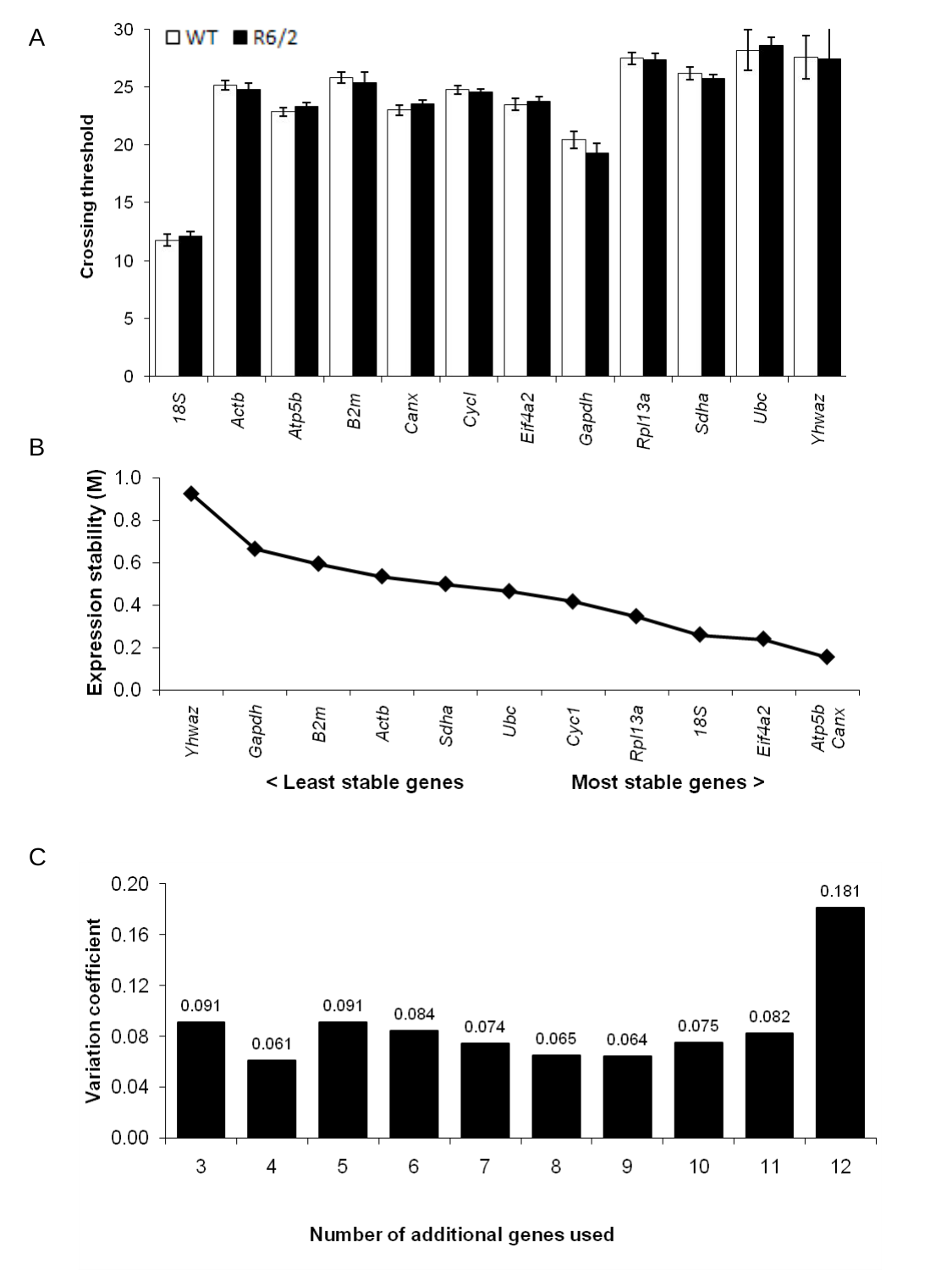

A
B
C

Supplement: Additional file 3 — GeNorm analyses to identify optimal reference genes in the cerebellum. (A) Raw crossing threshold (Ct) data for a panel of 12 potential references from the geNorm kit in wild-type (open bars) and R6/2 (filled bars) mice. Reference genes tested were 18S (18S ribosomal RNA subunit), Actb (beta-actin), Atp5b (ATP synthase subunit 5b), B2m (beta-2 microglobulin), Canx (calnexin), Cyc1 (cyclin D1), Eif4a2 (eukaryotic initiation factor 4a2), Gapdh (glyceraldehyde-3-phosphate dehydrogenase), Rpl13a (ribosomal protein L13a), Sdha (succinate dehydrogenase complex, subunit A), Ubc (ubiquitin C) and Yhwaz (phospholipaase A2). (B) Raw Ct data was subjected to analysis with the geNorm applet which automatically calculates the gene-stability measure M, which is an average pairwise variation of a particular gene with all other control genes. Therefore, genes with the lowest M value have the most stable expression, in this case across genotypes (ie comparing wild-type and R6/2 mice). Expression stability is plotted for each of the potential reference genes, progressing from the least stable genes with a higher M value to the most stable genes with a lower M value. (C) In order to measure expression levels accurately, normalization by multiple housekeeping genes is optimal. The graph illustrates the levels of variation in average reference gene stability with the sequential addition of each reference gene to the equation, starting with the most stably expressed genes on the left with the inclusion of a 4th gene etc, moving to the right. This measure is known as pairwise variation (V), the values of which are indicated above each bar. A V score of below 0.15 is the target. [file 1750-1326-3-17-S3.ppt]

## Slide 1
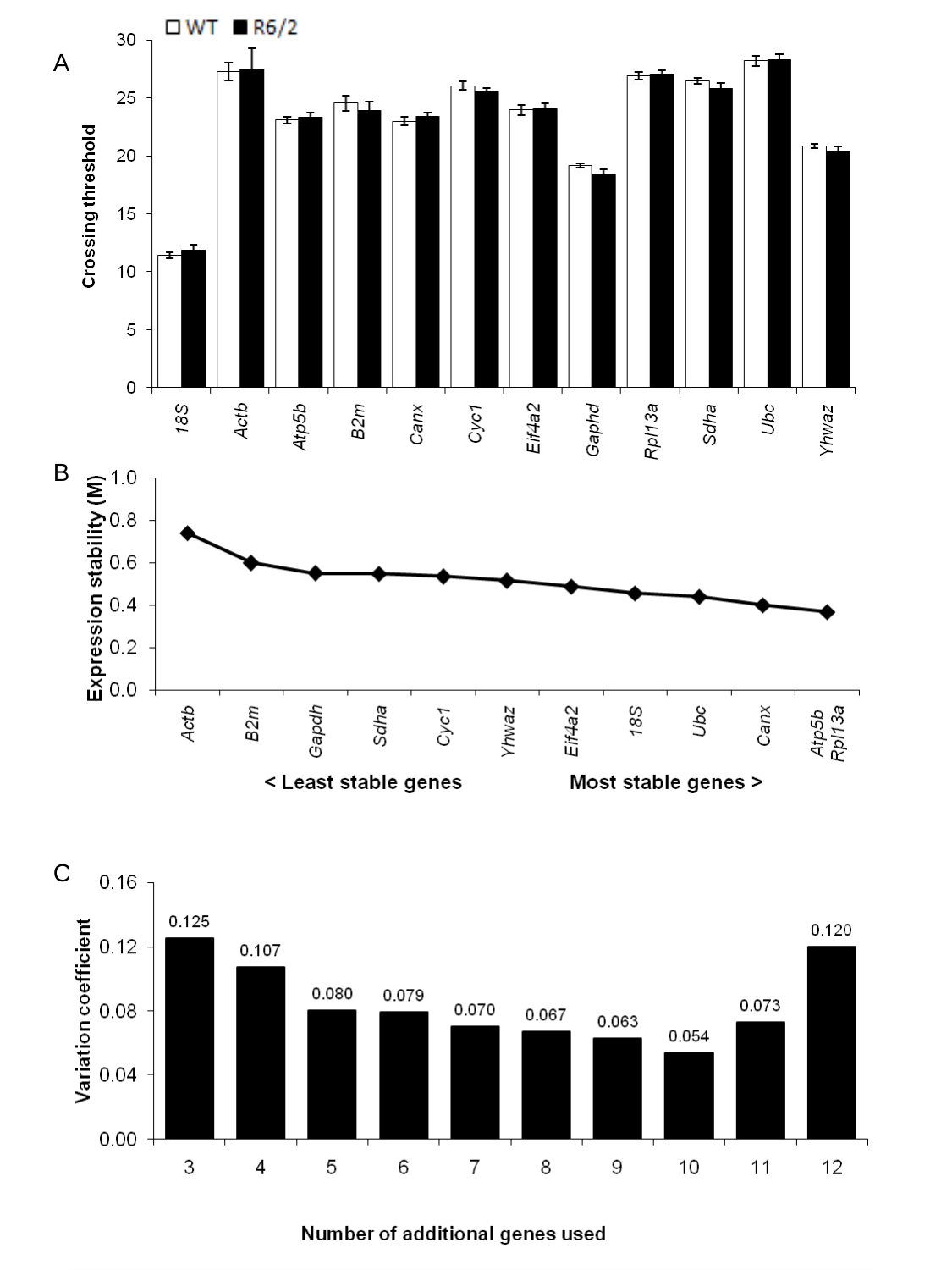

A
B
C

Supplement: Additional file 4 — GeNorm analyses to identify optimal reference genes in the cortex. (A) Raw crossing threshold data (Ct) for a panel of 12 potential references from the geNorm kit in wild-type (open bars) and R6/2 (filled bars) mice. Reference genes tested were 18S (18S ribosomal RNA subunit), Actb (beta-actin), Atp5b (ATP synthase subunit 5b), B2m (beta-2 microglobulin), Canx (calnexin), Cyc1 (cyclin D1), Eif4a2 (eukaryotic initiation factor 4a2), Gapdh (glyceraldehyde-3-phosphate dehydrogenase), Rpl13a (ribosomal protein L13a), Sdha (succinate dehydrogenase complex, subunit A), Ubc (ubiquitin C) and Yhwaz (phospholipaase A2). (B) Raw Ct data was subjected to analysis with the geNorm applet which automatically calculates the gene-stability measure M, which is an average pairwise variation of a particular gene with all other control genes. Therefore, genes with the lowest M value have the most stable expression, in this case across genotypes (ie comparing wild-type and R6/2 mice). Expression stability is plotted for each of the potential reference genes, progressing from the least stable genes with a higher M value to the most stable genes with a lower M value. (C) In order to measure expression levels accurately, normalization by multiple housekeeping genes is optimal. The graph illustrates the levels of variation in average reference gene stability with the sequential addition of each reference gene to the equation, starting with the most stably expressed genes on the left with the inclusion of a 4th gene etc, moving to the right. This measure is known as pairwise variation (V), the values of which are indicated above each bar. A V score of below 0.15 is the target. [file 1750-1326-3-17-S4.ppt]
