# Supplementary material for: Optimisation of region-specific reference gene selection and relative gene expression analysis methods for pre-clinical trials of Huntington's disease
Source: Mol Neurodegener. 2008 Oct 27;3:17. doi: 10.1186/1750-1326-3-17 (PMC2584034; doi:10.1186/1750-1326-3-17)
Supplement: Additional file 5 — Bdnf gene structure. This schematic of Mus musculus Bdnf gene structure (top panel, accession number AY057907) indicates the "classical" four promoters (blue boxes) and coding exon (white box) and the recently described additional promoters (yellow boxes) [39]. Only the coding exon is translated, giving rise to a protein of 289 amino acids. The NRSE site and intra-exonic splice sites on promoter II are also indicated. The bottom panel shows the splice variants arising from the Bdnf gene, including the splice variants arising from intra-exonic splice sites A, B and C in promoter II. [file 1750-1326-3-17-S5.ppt]

## Slide 1
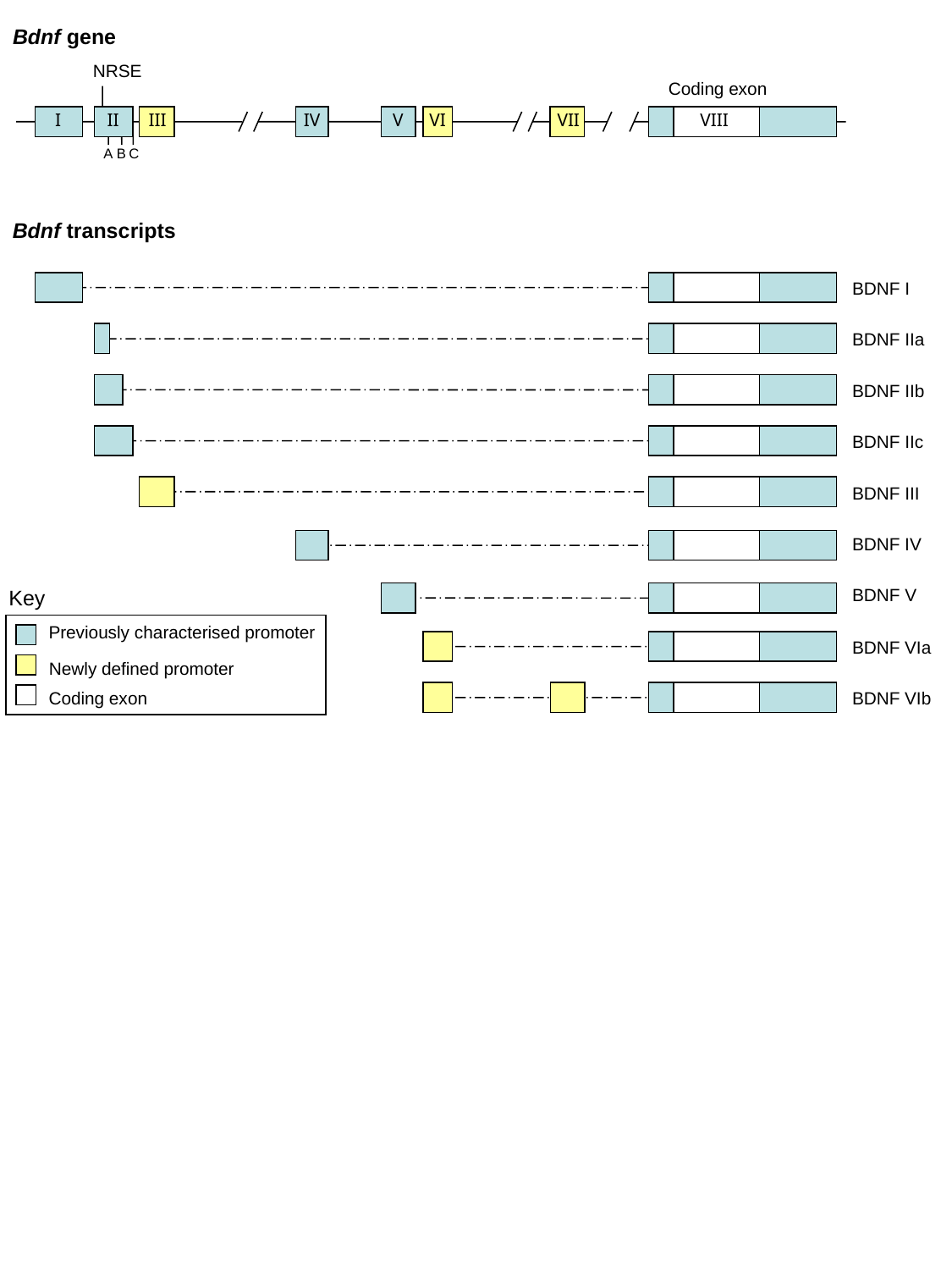

Bdnf gene
NRSE
I
II
III
IV
V
VI
VII
VIII
Coding exon
A
B
C
Bdnf transcripts
BDNF I
BDNF IIa
BDNF IIb
BDNF IIc
BDNF III
BDNF IV
BDNF V
Key
Previously characterised promoter
Newly defined promoter
Coding exon
BDNF VIa
BDNF VIb
