# Supplementary material for: Optimisation of region-specific reference gene selection and relative gene expression analysis methods for pre-clinical trials of Huntington's disease
Source: Mol Neurodegener. 2008 Oct 27;3:17. doi: 10.1186/1750-1326-3-17 (PMC2584034; doi:10.1186/1750-1326-3-17)
Supplement: Additional file 6 — Methods. Detailed methods [file 1750-1326-3-17-S6.doc]

# Detailed Methods: Supplementary Information

**RNA extraction**

QIAZOL (Qiagen) was added to frozen dissected brain regions (0.2 ml for striatum, 0.5 ml for cortex and cerebellum) prior to homogenisation. The homogenate was flash-frozen and stored at -80^o^C until all samples are prepared. The homogenate was thawed and placed at room temperature for five minutes to promote dissociation of nucleoprotein complexes. An equal volume of chloroform was added prior to thourough mixing. The homogenate was incubated at room temperature for 3 minutes then centrifuged at 13,000 x *g* for 15 min at 4°C. The upper aqueous phase was transferred to a new eppendorf and one volume of 70% ethanol was added, then mixed thoroughly. 700μl of sample was transferred to a RNeasy Mini spin column (Qiagen) in a 2ml collection tube. The column/tube assembly was centrifuged for 15 sec at maximum speed at room temperature. The flow-through was discarded and the centrifugation step was repeated with remaining solution from the previous step. 350μl Buffer RW1 was added to the RNeasy spin column and centrifuged for 15 sec at max speed at room temperature prior to discarding the flow-through. DNase treatment was performed using Qiagen RNase-Free DNase Set. For each sample 10μl DNaseI stock solution (lypholised DNase was reconstituted in 550μl RNase free water, aliquoted & stored at -20°C) was diluted in 70μl RDD buffer (i.e. 1:8 dilution), and mixed gently by inverting the tube. 80μl of the DNaseI mix was added to each RNeasy spin column membrane, and incubated at room temperature for 15 min. The washes were performed by adding 350μl Buffer RW1 to the RNeasy spin column prior to centrifuging for 15 sec at max speed at room temperature and discarding the flow-through. 500μl Buffer RPE (with ethanol) was added to the RNeasy spin column, centrifuged for 15 sec at max speed at room temperature and the flow-through was discarded. The step was repeated with a further 500μl Buffer RPE.

The RNeasy spin column was placed in new 2ml collection tube & centrifuged for 15 sec at max speed at room temperature to remove any residual ethanol. The RNeasy spin column was placed in a new 1.5ml collection tube and 50μl RNase-free water was added directly to the membrane and incubated at room temperature for 1 min. The column assembly was centrifuged for 15 sec at max speed at room temperature. The elution step was repeated using either another volume of RNase-free water (for cortex or cerebellum) or re-using both the eluate the collection tube (for striatum). The RNA was stored at -80°C (can store at -20°C for short periods if necessary). The RNA was quantified using an Agilent Bioanalyser according to the manufacturer’s instructions prior to cDNA synthesis.

**Reverse transcriptase (RT)**

All experimental samples were prepared in parallel, adding the same amount of RNA template to each RT reaction, using the same premixes. Total RNA (either 5 μg of RNA from cerebellum and cortex or 1 μg of striatal RNA) was diluted in a volume of 9 μl of DEPC-treated water. In order to set up the RT reactions, 3 ul of Premix 1 was added to 9 μl of each sample:

Reagent

DTT 0.1M 2ul (Final concentration 10 mM)

Random hexamers 100ng/ul 1ul (Final concentration 10 ng/μl)

The mix of RNA, DTT and random oligonucleotide hexamers was incubated in RT94 programme (94^o^C for two minutes) and then cooled on ice for 2 minutes. In the meantime, Premix 2 was prepared:

Reagent

DEPC H2O 0.75ul

10mM dNTPs 2ul (Final concentration 1 mM)

5x 1^st^ Strand buffer 4ul

RNasin (Promega) 0.25ul (Final concentration 0.35 U/μl)

MMLV-RT (Invitrogen) 1ul (Final concentration 14 U/μl)

1^st^ Strand buffer consisted of 50 mM KCl, 10 mM Tris–HCl (pH 9.0), 0.1% Triton X-100, 6.5 mM MgCl_2_.

8 μl of Premix 2 was aliquoted into each sample tube bringing up the total volume to 20 μl prior to incubation in RT3Temp programme (10 minutes at 23°C, 40 minutes at 37°C followed by 5 minutes at 94^o^C to inactivate the enzyme). The cDNA samples were then cooled to 4^o^C and diluted 1:10 (to 200 μl) before storing at -20^o^C.

**Quantitative PCR (qPCR)**

qPCR reactions were performed in triplicate with 5 μl of diluted cDNA template in a 25 μl reaction containing 2x Precision Mastermix (PrimerDesign), primers and probe using the Opticon 2 and Chromo4 real-time PCR machines (Biorad). Three types of reactions were set up: (1) PrimerDesign housekeeping gene assays using the Perfect Probe system, (2) Gene of interest assays using primers and Taqman dual-labeled probes (5’ FAM and 3’ TAMRA) and (3) Gene of interest assays using primers and SYBR-green supplemented Precision MasterMix.

*1) Set up PrimerDesign HK qPCR for housekeeping genes*

Component 1 Reaction

Reconstituted primer/probe mix 1 μl* (300nM Perfect Probe primer)

2X qPCR Mastermix 10 μl

PCR-Grade water 4 μl

Final volume 20 μl

15 μl of the mastermix was pipetted into each well according to the plate set up prior to adding 5 μl of diluted cDNA template (or no-template controls). Real-time PCR amplification was performed using the HKGene programme (10 min at 95^o^C followed by 40 cycles of 15 sec at 95^o^C and 30 sec at 50^o^C).

*2) Set up qPCR for genes of interest using Taqman*

Component 1 Reaction

2X qPCR Mastermix 12.5 μl

Forward primer (10uM) 0.75 ul (300 nM final concentration)

Reverse primer (10uM) 0.75 ul (300 nM final concentration)

Probe (10uM) 5’6-FAM, 3’TAMRA 0.5 ul (200 nM final concentration)

PCR-Grade water 5.5 μl

Final volume 25 μl

20 μl of the mastermix was pipetted into each well according to the plate set up prior to adding 5 μl of diluted cDNA template (or no-template controls). Real-time PCR amplification was performed using the Taqman programme (15 min at 95°C followed by 40 cycles of 15 sec at 95°C and 1 min at 50°C).

*2) Set up qPCR for genes of interest using SYBR green*

Component 1 Reaction

2X Mastermix with SYBRgreen 12.5 μl

Forward primer (10uM) 0.75 ul (300 nM final concentration)

Reverse primer (10uM) 0.75 ul (300 nM final concentration)

PCR-Grade water 6 μl

Final volume 25 μl

20 μl of the mastermix was pipetted into each well according to the plate set up prior to adding 5 μl of diluted cDNA template (or no-template controls). Real-time PCR amplification was performed using the SYBRgreen programme (2 min at 50°C, 15 min at 95°C followed by 40 cycles or 15 sec at 94°C and 1 min at 60°C).

**GeNorm analysis**

We used the geNorm Housekeeping Gene Selection Kit (PrimerDesign) to evaluate 12 commonly used housekeeping genes in 3 different dissected brain regions from wild-type and R6/2 mice. For the 12 gene kit, we used 132 μl of high-quality cDNA extracted from 12 week R6/2 and wild-type striatum at a concentration of 5 ng/μl. In the meantime, each lyophilised primer-probe mix supplied from PrimerDesign was resuspended in 220 μl of water and mixed thoroughly. A premix was made up for each reference gene as follows:

Component 1 Reaction

Reconstituted primer/probe mix 1 μl* (300nM final concentration)

2X qPCR Mastermix 10 μl

PCR-Grade water 4 μl

15 ul of the mix was added to each well according to the plate set up. 5 ul of each sample and negative no-template controls were then added, with samples being run in triplicate. All samples for each reference were run together on the same plate, with one plate per reference gene. All plates were prepared at the same time in order to reduce variability between plates. Plates were run on the machine using the GeNorm programme (10 min at 95^o^C followed by 50 cycles of 15 sec at 95^o^C, 30 sec at 50^o^C with fluoregenic data collected through the FAM channel, then 15 sec at 72^o^C).

The C_t_ values for each sample were averaged to obtain raw C_t_ values in Microsoft Excel and transformed into relative quantification data using the deltaC_t_ method. To perform deltaC_t_ analyses, we first subtracted the highest C_t_ value from all other C_t_ values for each gene measured such that the highest value was 0 and all other values were less than 0. Then for each data point, we applied the equation; 2^(-delta Ct)^. This expressed all data relative to the expression of the least expressed gene. The transformed data was used to prepare an input file for geNorm analyses in Excel with the first column containing the sample names and the first row containing the gene names. The first cell of the first row and column (cell A1) were left empty and the file was saved in the InputData directory provided by PrimerDesign. All running instances of Excel were closed and the geNorm applet was started (provided by PrimerDesign, with macros enabled. The geNorm applet is also available freely on the web: [http://www.gene-quantification.de/hkg.html#genorm](http://www.gene-quantification.de/hkg.html#genorm). The input file was loaded in geNorm and the automated analysis was enabled which generated two geNorm charts as an output. The first chart ranks the candidate reference genes according to their expression stability and the second determines the number of reference genes required for optimal normalisation, expressed as pairwise variation (V).

**Relative quantification of qRT-PCR data**

In order to perform relative quantification, all C_t_ values must be transformed into relative quantification data, expressed as 2^-ΔΔCt^. Crossing threshold data can be imported into Excel from real-time PCR platforms and subsequent analyses can be performed in Excel. During 2^-ΔΔCt^ analysis, each sample (run in triplicate) was treated separately. The mean C_t_ of each sample and standard deviations were calculated. Standard deviations should be within 1 C_t_, and if there were outliers (> 2 standard deviations), these data points were removed from the analysis. The means of each sample were used for analysis. The first step of analysis (ΔC_t_) was to normalise all the samples with respect to the least expressed sample, thus subtracting the highest C_t_ value from the C_t_ value of each sample. In this step, the least expressed sample had a ΔC_t_ value of 0 and all the other samples had negative values. Hence all data was expressed relative to the expression of the least expressed gene. The second step was to perform the function (ΔC_t sample_ – ΔC_t reference_), which gives a ΔΔC_t_ value. The third step was to calculate 2^-ΔΔCt^, which yielded the expression ratio (as a positive integer) for each individual sample. After performing the 2^-ΔΔCt^ calculation for each sample, we calculated the means for each group in addition to determining the standard deviation and standard error of the mean (SEM) thus providing a relative expression ratio (in arbitrary units) and variation between ratios. The expression ratio data were amenable to statistical tests such as analysis of variance (ANOVA) to the expression ratios.

**Statistical analysis**

Preliminary statistical analyses (Student’s *t*-test and power calculations were performed using Excel. Independent samples *t* test was performed using SPSS in order to confirm the Student’s *t*-test results.
